# Response of Vegetation and Soil Carbon and Nitrogen Storage to Grazing Intensity in Semi-Arid Grasslands in the Agro-Pastoral Zone of Northern China

**DOI:** 10.1371/journal.pone.0096604

**Published:** 2014-05-12

**Authors:** Min-yun Xu, Fan Xie, Kun Wang

**Affiliations:** 1 Department of Ecology, College of Urban and Environmental Sciences, Peking University, Beijing, P.R. China; 2 College of Animal Science & Technology, Hebei Agricultural University, Baoding, Hebei province, P.R. China; 3 Department of Party Affairs, China National Light Industry Council, Beijing, P.R. China; 4 College of Animal Science & Technology, China Agricultural University, Beijing, P.R. China; University of Maryland, United States of America

## Abstract

Overgrazing has been the primary cause of grassland degradation in the semi-arid grasslands of the agro-pastoral transition zone in northern China. However, there has been little evidence regarding grazing intensity impacts on vegetation change and soil C and N dynamics in this region. This paper reports the effects of four grazing intensities namely un-grazed (UG), lightly grazed (LG), moderately grazed (MG) and heavily grazed (HG) on vegetation characteristics and soil properties of grasslands in the Guyuan county in the agro-pastoral transition region, Hebei province, northern China. Our study showed that the vegetation height, canopy cover, plant species abundance and aboveground biomass decreased significantly with increased grazing intensity. Similarly, soil organic carbon (SOC) and total nitrogen (STN) in the 0–50 cm were highest under UG (13.3 kg C m^−2^ and 1.69 kg N m^−2^) and lowest under HG (9.8 kg C m^−2^ and 1.22 kg N m^−2^). Soil available nitrogen (SAN) was significantly lower under HG (644 kg N hm^−2^) than under other treatments (725–731 kg N hm^−2^) in the 0–50 cm. Our results indicate that the pasture management of “take half-leave half” has potential benefits for primary production and livestock grazing in this region. However, grazing exclusion was perhaps the most effective choice for restoring degraded grasslands in this region. Therefore, flexible rangeland management should be adopted in this region.

## Introduction

Grazing, the most common use of grasslands, can influence plant community structure, soil properties and nutrient cycling within the plant-soil system[1]. For instance, selective grazing caused changes in plant species composition[2] and influenced herbage production[3]. Several previous studies proposed that light and moderate grazing favored grasses and stimulated grassland productivity[4] [5]. However, increasing grazing intensity, to some extent, generally decreased grassland productivity, canopy height and sward cover[6], but increased unpalatable species proportion[4], and destroyed soil aggregation[7]. Milchunas and Lauenroth[2] analyzed 236 grazing studies worldwide and found different results with dynamics of species composition, root biomass, soil organic C and soil N of grasslands not closely associated with grazed or non-grazed measurements. These contradictory findings suggested that the effects of grazing on nutrient cycling and ecosystem functioning still needs further study [2].

Soil water content was the most limiting factor for primary productivity in semi-arid rangelands[8]. Grazing intensity affected soil water conditions of grasslands through animal trampling and intake behavior. Trampling compacted soil and increased soil bulk density[9], which led to the reduction of storage capacity and supply of soil water and nutrients, ultimately decreasing soil fertility and grassland productivity[10]. Naeth et al.[11] reported that soil water in grazed treatments was generally lower than in non-grazed grassland. However, grazing could have the opposite effect on soil water through removing vegetation, resulting in decreased evapo-transpiration [11].

With regard to soil C and N, previous investigations have produced very variable results. Grazing management usually has a negative effect [1] [9] [12] on soil C storage, with well-managed grazing was reported to enhance C sequestration [13] [14]. However, Milchunas and Lauenroth[2] found that grazing management had no effect on soil C and N storage. In addition, Frank et al.[1] found heavy grazing did not reduce soil C but moderately grazed pasture contained less soil C compared to a non-grazed exclosure, which was attributed to changes in species composition. Bauer et al.[15] found greater total N content to a soil depth of 0.457 m in grazed than non-grazed grasslands. Schuman et al. [16] found 12 years of grazing under different stocking rates did not change the total C and N mass of in the top 60 cm of the plant-soil system, but changed the distribution of C and N among the system components, primarily via a significant increase in the of C and N mass in the root zone (0–30 cm) of the soil profile. Wu et al.[17] reported that the temperate grasslands of northern China could achieve significant C and N storage on decadal scales in the context of mitigating global climate change by grazing elimination. He et al.[18] found that C and N storage in both the 0–10 cm and 10–30 cm soil layers decreased linearly with increasing stocking rates in northern China. They also found there existed an underlying transformation from soil C sequestration under light grazing to C loss under heavy grazing, which implied that grasslands used for grazing have the capacity to sequester C in the soil under appropriate grazing pressure[18]. These varied findings were likely associated with the differences in climate, soil properties, study sites, plant community composition and grazing management measurements [19], indicating that more studies are needed to further clarify C and N dynamics under different grazing intensities and grazing regions.

Scientists have already conducted numerous studies on the effects of grazing pressure on plant and soil properties of grasslands in northern China, but most have been in the pastoral zone [12] [20]. Relatively little research has focused on the productivity and sustainability of grasslands in agro-pastoral areas[21]. The agro-pastoral transition zone in semi-arid northern China [22], is one of the world's largest agro-pastoral ecotones, with an area of 69×10^4^ km^2^ and a population of approximately 6.7×10^7^ [23]. Grasslands in this area play an important role in providing ecological services, such as sequestering carbon, regulating nutrient cycling, affecting biodiversity and ecosystem functioning [24]. Overgrazing and land use changes that reduces the areas of forests and grasslands along with an increase in agricultural activity have intensified pressures on the regional grasslands, causing severe land degradation[25]. Therefore, it is very important to understand the effects of grazing intensity on plant characteristics and soil properties of grasslands in this region. The objectives of this study were to quantity the effects of grazing intensity on (i) biomass allocation patterns and vegetation characteristics, (ii) soil properties and C and N storage dynamics in soil, and (iii) the relationship between soil C and N storage and the grazing-induced variation in plant biomass allocation patterns.

## Materials and Methods

### Study sites

**Figure 1:**
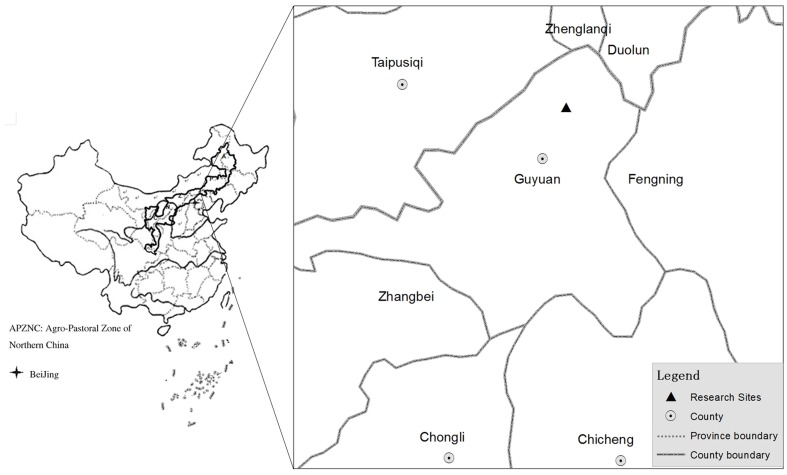
Location of the research sites in the agro-pastoral zone of northern China.

The research was conducted at the Guyuan Grassland Ecosystem Observation and Research Station of China Agricultural University (115°41′E, 41°46′N), in Hebei province, northern China (Figure 1). With elevation between 1300 to 1450 m a.s.l., this area belongs to a semi-arid agro-pastoral transitional region, with a typical continental climate. The mean annual precipitation and potential evaporation range from 350 to 450 mm and 1700 to 2300 mm, respectively (1990 to 2010), with 80% of precipitation occurring during the June through September growing season. The mean annual temperature ranges from 1 to 2°C (1990 to 2010). The region is characterized by open, flat land, alternating with undulating hilly land. Further details of physiographic characteristics of this region have been presented by Chen and Zhu[26]. Soils are classified as chestnut type soils (i.e. Calcic Kastanozems), which are equivalent to Calcic-orthic Aridisol in the US soil taxonomy classification system. Soil pH ranges from 7.79 to 8.49 [27]. The plant community is mainly dominated by *Leymus chinensis, Stipa grandis, Cleistogenes squarrosa, Agropyron michnoi*, and *Koeleria cristata* [28].

### Experimental design

In this study, four contiguous sites were selected based on the availability of reliable information on the grassland management history under the same environmental conditions. Prior to the establishment of the Guyuan Grassland Ecosystem Observation and Research Station in 2000, the area had not been grazed or only lightly grazed by domestic livestock for about 20 years. These sites had been exposed to a 10-year controlled grazing experiment (2001 to 2011) using different grazing pressures by herds consisting of 70 to 80% goats and sheep and 20 to 30% Holstein and beef cattle. Stocking rate was the basis for the four grazing intensities used, un-grazed (UG), lightly grazed (LG), moderately grazed (MG) and heavily grazed (HG). Calculation of the stocking rate is defined in terms of number of sheep units/pasture area, where a sheep unit (in China) is defined as a 40 kg liveweight ewe nursing lamb(s) with daily forage consumption of 5 to 7.5 kg[29]. Based on their forage intake and this definition of a sheep unit, ten goats were equal to eight sheep, and five sheep were approximately equivalent to one Holstein or beef cow. Animals were kept in pens at night, so the input of faecal organic material was reduced. There was no application of additional fertilizers to the four experimental sites.

The UG grassland was a 10 ha area with no grazing or human disturbance for livestock production. The vegetation was dominated by *Leymus chinensis*, *Stipa grandis* and *Hordeum spontaneum*. The LG grassland carried 11.16 sheep units within a 12.0 ha area, and its stocking rate was 0.93 sheep units per ha, resulting in 20% grassland utilization. The vegetation was dominated by *Leymus chinensis, Potentilla lancinata*, and *Carex capricornis*. The MG grassland supported 74.52 sheep units within a 32.0 ha area, and its stocking rate was 2.33 sheep units per ha, resulting in 50% grassland utilization. The vegetation was dominated by *Leymus chinensis, Thermopsis lanceolata, Ixeris polycephala, Puccinellia altaica Tzvel* and *Iris lactea var. pall. chinensis (Fish) Koidz*. The HG grassland supported 114.50 sheep units within a 35.12 ha area, and its stocking rate was 3.26 sheep units per ha, resulting in 70% grassland utilization. The vegetation was dominated by *Leymus chinensis, Iris lactea var. pall. chinensis (Fish) Koidz* and *Stellera chamaejasme*.

### Sampling and field investigation

In each of the four experimental sites, three sampling plots (50 m×50 m) were selected with the constraint that they were at least 2.0 m from the margin to avoid any edge effects. In each sampling plot, three sampling quadrats were randomly selected using a 0.5×0.5 m metal frame along the diagonal gradients. The experiment comprised 36 quadrats (3 plots x 3 quadrats x 4 grazing intensities). The vegetation composition survey was performed during the peak standing biomass in early September of 2012. It included measurements of plant canopy cover, mean vegetation height, aboveground biomass (comprising live biomass, standing dead material and litter on the soil surface), and plant species abundance across the four grazing intensities. Plant species composition was estimated using three sampling quadrats along a 10 m line intercept located at the centre of each plot. Plant canopy cover was determined using point frames (a 0.5 m×0.5 m metal frame with 50 grids) combined with a visual estimation method. The standing crop was harvested near ground-level using hand shears. Surface litter and standing dead plant biomass were hand brushed to bare the sampling area after herbage was clipped. The material was oven-dried at 65°C to a constant weight.

To estimate root biomass, five soil cores were collected at 10 cm intervals to 50 cm depth using a soil core sampler (6 cm diameter) from each quadrat, and placed in mesh bags, then rinsed in water to remove soil and debris. The root samples were dried at 80°C to a constant weight. Final biomass estimates were converted to a kilogram per hectare basis using the area of the sample quadrat, or the surface area in the case of the roots.

For soil C and N analysis, five soil samples were collected from three quadrats in each sampling plot after the aboveground material was harvested. Soil samples were collected using a soil core sampler (6 cm diameter) and separated into 0–10, 10–20, 20–30, 30–40, and 40–50 cm increments. Five cores were taken from each layer and combined to provide an adequate sample for analyses. Samples were placed in sealed plastic bags in the field, then air-dried in a ventilated room, ground, then sieved to <2 mm to remove stones, root fragments and organic debris before chemical analyses. Soil samples were analyzed for organic C by the external heating method[30]. Soil total nitrogen (STN) content was determined using the Kjeldahl acid-digestion method with an auto-analyzer (Foss Inc., FIAstar5000, Sweden). Soil available nitrogen (SAN) was measured by the methods of Page et al.[31]. Soil bulk density was measured using samples taken with a volumetric (100 cm^3^) steel ring and calculated as the mass of oven-dry soil (105°C) divided by the core volume [32] for each depth increment. The measurement of bulk density allows the estimation of the total soil C and N storage under different grazing intensities. Soil water content was determined by weighing samples before and after oven-drying at 105°C for 8 h.

### Derived Variables Calculations

Soil organic C (SOC, kg C m^−2^), STN (kg N m^−2^), and SAN (kg N hm^−2^) were calculated on an area basis to a soil depth of 50 cm as follows: 
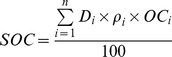
(1)

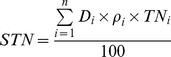
(2)

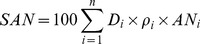
(3)where i is the number of soil horizons, D_i_ is the depth interval (cm) of the horizon i from the top soil down, ρ_i_ is the soil bulk density (g cm^−3^) in the horizon i, OC_i_ is the mean SOC content (g kg^−1^) in the horizon i, TN_i_ and AN_i_ represent total N concentration (g kg^−1^) and available N concentration (mg kg^−1^) respectively.

### Data analysis

All statistical analyses were conducted using SPSS 16.0 for Windows (SPSS Inc., Chicago, IL, USA). Graphical presentation was done with SigmaPlot 10 (SPSS Inc., Chicago, IL, USA) and Adobe Acrobat XI Pro (Adobe Systems Incorporated, California, USA). Statistical analysis of each measured variable was conducted with the GLM (general linear model) procedure. One-way analyses of variance (ANOVA) were conducted to assess treatment effects on grassland vegetation characteristics, soil C and N storage properties. Individual system components (litter, standing dead and live biomass, roots by depth and soil by depth) were tested with a separate analysis of variance with replicate pastures treated as blocks. Error variances for depths were heterogeneous, so only univariate analysis was reported for depth. Least-significant-differences (LSD) procedures were used for comparisons between means. Normal distribution and homogeneity of variance for each collective were tested using the Shapiro-Wilk's test and Levene's test. Significant differences for all of the statistical tests were evaluated at the level of P = 0.05.

## Results

### Vegetation characteristics

Our study demonstrated that the vegetation height, canopy cover, plant species abundance and aboveground biomass (live, standing dead and litter biomass) of grasslands in the agro-pastoral zone, decreased significantly with increased grazing intensity over 10-years (Table 1).

**Table 1:** Vegetation height, canopy cover, plant species abundance, aboveground biomass, belowground biomass, and R:S (root/shoot) ratio of grasslands under different grazing intensities in agro-pastoral zone.

System components	UG	LG	MG	HG
Vegetation height (cm)	20.23±1.10a	17.10±0.99b	10.61±0.94c	5.68±0.87d
Canopy cover (%)	82.78±1.12a	78.44±1.03a	63.89±2.34b	40.89±1.82c
Species abundance (species m^−2^)	41.67±1.94a	30.44±1.60b	33.89±1.52b	29.44±1.84b
Aboveground biomass (g m^−2^)	575.79±5.68a	462.30±6.20b	439.51±5.18b	161.88±4.95c
Live biomass	538.80±5.56a	436.13±6.26b	413.50±5.17b	149.36±4.93c
Standing dead biomass	21.92±0.28a	14.31±0.34b	13.39±0.23b	5.40±0.21c
Surface litter biomass	15.08±0.33a	11.96±0.17b	12.62±0.19b	7.11±0.17c
Belowground biomass (g m^−2^)	1297.38±4.14c	1599.42±4.34b	1694.09±4.62a	1277.04±11.05c
0–10 cm	940.90±6.17d	1162.92±8.64b	1277.66±7.15a	1026.85±8.69c
10–20 cm	162.17±1.99b	183.98±3.70a	186.13±2.42a	130.80±1.56c
20–30 cm	105.78±2.76b	128.04±2.19a	136.14±2.85a	57.08±0.64c
30–40 cm	62.61±2.36c	75.66±1.17a	68.16±2.11b	36.78±1.85d
40–50 cm	25.92±1.07b	48.81±1.03a	26.30±0.66b	25.53±2.23b
Total plant biomass (g m^−2^)	1873.18 ±4.79b	2061.72±8.12a	2133.61±7.70a	1438.92±10.80c
R:S	2.32±0.03c	3.57±0.05b	3.97±0.05b	8.32±0.39a

Note: UG, LG, MG, and HG are four grazing intensities: un-grazed; lightly grazed; moderately grazed; and heavily grazed. Values in the table are means ± SE (standard error). Different letters across the same component indicate significant difference for the different grazing intensities (p<0.05).

The UG plots had significantly higher plant species abundance than the other three plots with, the MG pasture displaying a similar species abundance to the LG and HG pastures. Our research also showed that increased grazing significantly decreased the canopy cover, with no significant difference between the UG and LG plots. The UG plots had significantly higher aboveground biomass, and HG plots had significantly lower value, while LG and MG plots showed intermediate values that were not significantly different. Belowground biomass of the MG plots was significantly higher than the other plots. Surprisingly, root biomass of the UG and HG pastures were similar and significantly lower than that of the LG pasture. This change was reflected in the higher root to shoot biomass ratios under the heavy grazing treatment compared to the light grazing treatment (Table 1). The total plant biomass in the LG and MG plots were similar, which were higher than those in the UG and HG plots. The HG plots had the lowest total biomass among the four grazing plots.

### Soil moisture content

Increasing grazing intensity generally decreased soil moisture content (Table 2). The overall trend was that the mean soil moisture content was highest in the UG plots and lowest in the HG plots in the upper horizons (0–40 cm), and there was no significant difference in the 40–50 cm soil layer.

**Table 2:** Soil moisture content and soil bulk density in grasslands to 50-pastoral zone.

Parameters	Depth intervals (cm)	Grazing intensities			
		UG	LG	MG	HG
Soil moisture content (%)	0–10	18.03±0.24a	16.58±0.35b	15.95±0.29b	10.66±0.23c
	10–20	20.09±0.27a	18.98±0.14b	19.23±0.22ab	14.60±0.42c
	20–30	20.72±0.43a	18.06±0.18b	17.56±0.32b	17.35±0.30b
	30–40	18.25±0.33a	17.47±0.47a	15.45±0.25b	15.66±0.30b
	40–50	18.85±0.23a	17.97±0.34a	18.26±0.30a	17.87±0.34a
Soil bulk density (g cm^−3^)	0–10	1.05±0.03c	1.20±0.03b	1.23±0.01b	1.47±0.01a
	10–20	1.38±0.01a	1.37±0.02a	1.40±0.03a	1.37±0.01a
	20–30	1.47±0.01a	1.45±0.01a	1.46±0.02a	1.44±0.02a
	30–40	1.46±0.01a	1.47±0.01a	1.47±0.02a	1.47±0.03a
	40–50	1.56±0.02a	1.55±0.02a	1.54±0.03a	1.61±0.02a

Note: UG, LG, MG, and HG are four grazing intensities: un-grazed; lightly grazed; moderately grazed; and heavily grazed. Values in the table are means±SE (standard error). Difference letters within each row indicate significant difference for the different grazing intensities (p<0.05).

### Soil bulk density

Soil bulk density increased with each soil depth increment for each grazing intensity (Table 2). The changes in soil bulk density due to grazing were significant in the 0–10 cm soil layer among the different stocking rates. However, grazing had no effect on the bulk density of the lower horizons (10–50 cm) among the four grazing intensities.

### SOC, STN, and SAN concentrations

**Figure 2:**
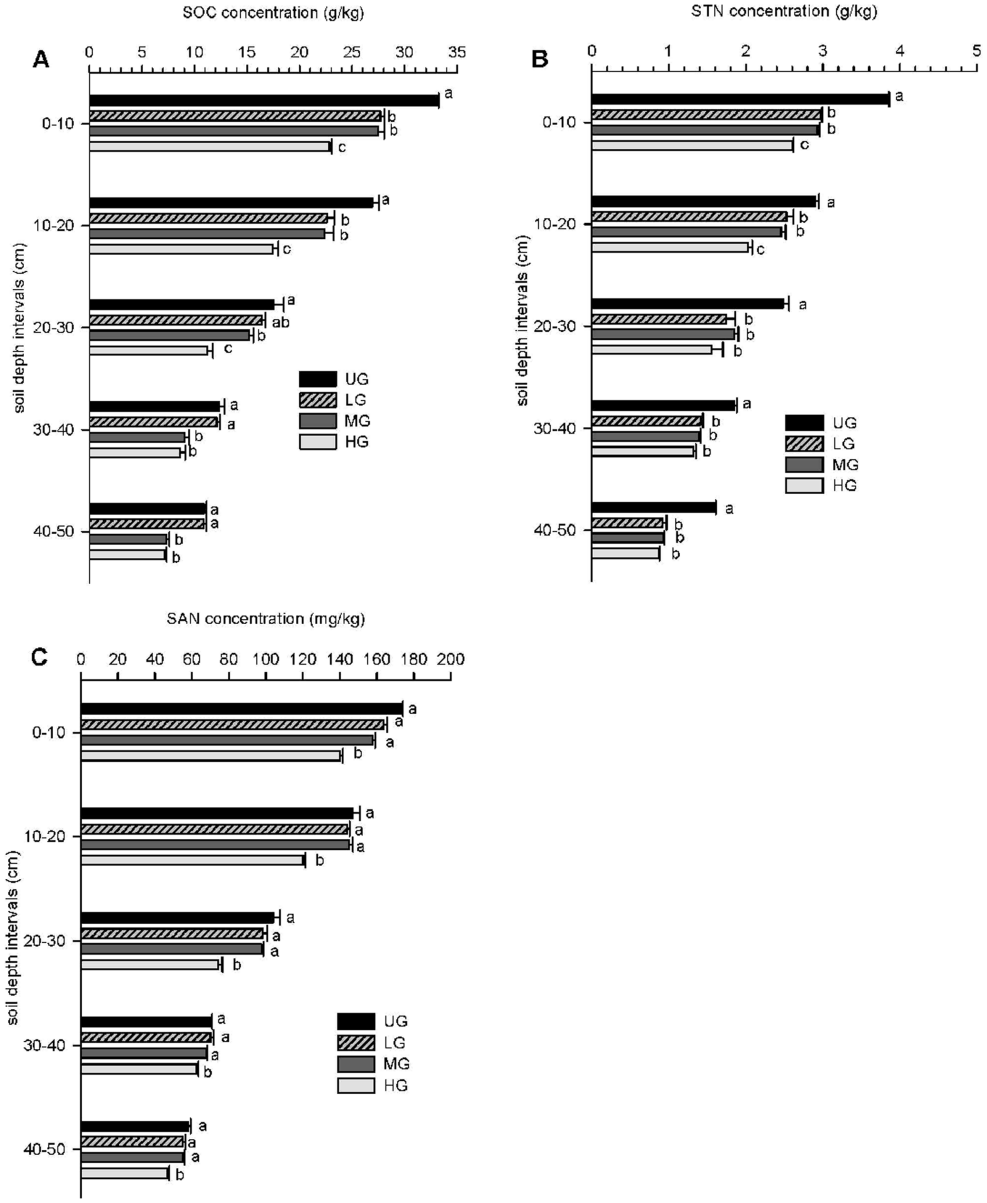
Concentration of SOC (A), STN (B), and SAN (C) under different grazing intensities. Soil organic carbon (SOC), soil total nitrogen (STN) and soil available nitrogen (SAN) concentrations in grasslands measured at 10 cm increments to depths of 50 cm in heavily grazed (HG), moderately grazed (MG), lightly grazed (LG), and un-grazed (UG) plots in the agro-pastoral zone. Within soil depth intervals, different letters indicate treatments are significantly different (p<0.05). Horizontal bars show S.E. n = 9.

For SOC, the UG plots had significantly higher values, and the HG plots significantly lower values in the 0–10, 10–20 and 20–30 cm soil layers, while the LG and MG plots showed intermediate values that were not significantly different. In the 30–40 and 40–50 cm soil layers, the UG and LG plots had similar values that were significantly higher than the MG and HG plots, which did not differ. In all four plots, SOC decreased with successive soil depth increments (Figure 2A).

The STN values in the UG plots were significantly higher than the other three plots across all of the 0–50 cm soil layers. In the 0–10 and 10–20 cm soil layers, HG had the lowest STN (2.60 and 2.03 g kg^−1^), and there was no difference between the LG (2.97 and 2.53 g kg^−1^) and MG (2.93 and 2.46 g kg^−1^) plots. In the 20–30, 30–40 and 40–50 cm soil profiles, STN in LG, MG, and HG did not differ (Figure 2B). SAN in the HG plots was significantly lower than in the UG, LG, and MG plots across all the 0–50 cm soil layers (Figure 2C). STN and SAN were all higher in the surface soil and decreased with soil depth among all four grazing intensities.

### SOC, STN, and SAN storage

**Figure 3:**
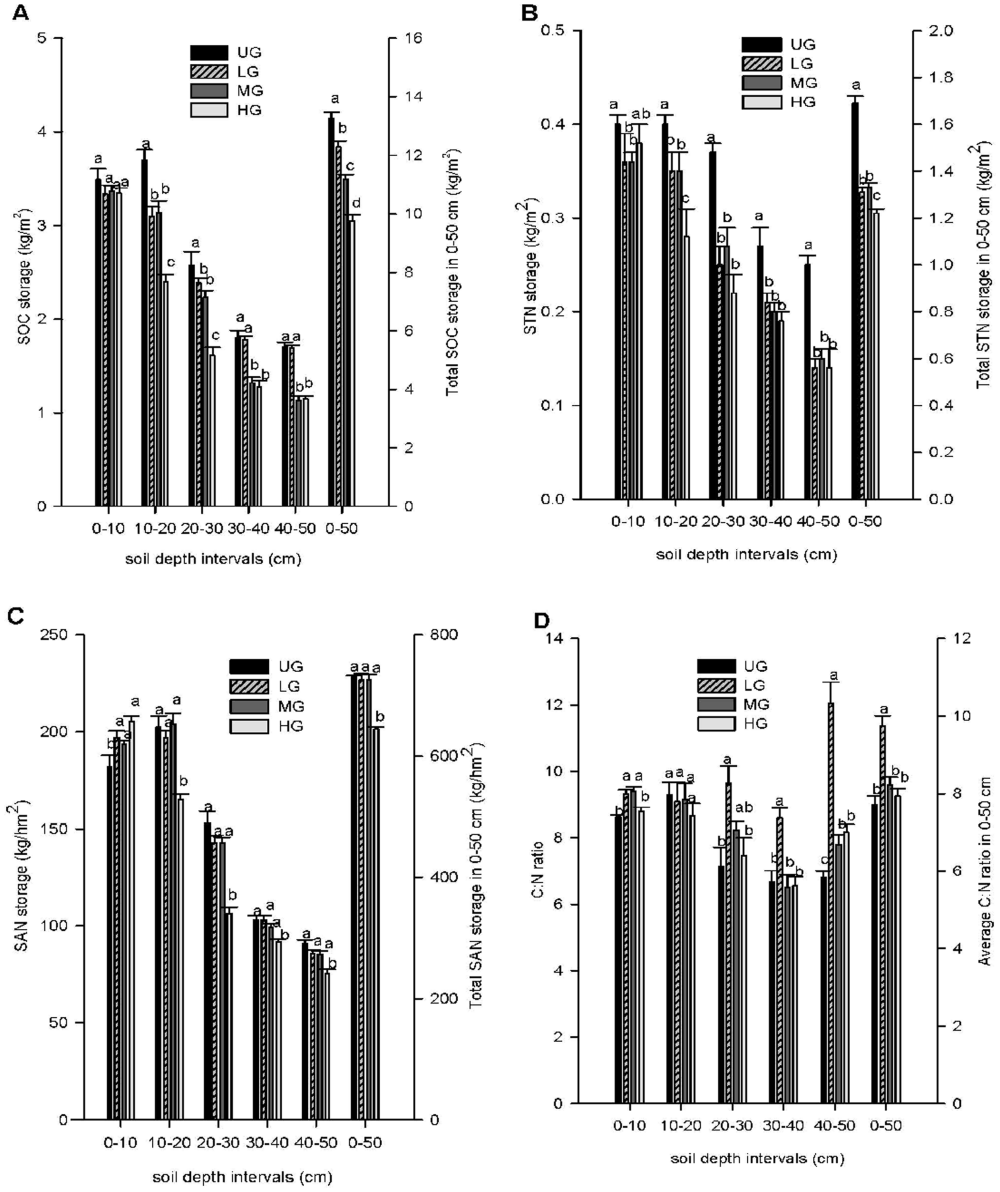
SOC (A), STN (B), SAN (C) storage and C:N ratio (D) under different grazing intensities. Soil organic carbon (SOC), soil total nitrogen (STN), soil available nitrogen (SAN) storage and C:N ratio in grasslands measured at 10 cm increments to depths of 50 cm in heavily grazed (HG), moderately grazed (MG), lightly grazed (LG) and un-grazed (UG) in the agro-pastoral zone. Within soil depth intervals, different letters indicate treatments are significantly different (p<0.05). Vertical bars show S.E. n = 9.

Trends in the storage of SOC, STN, and SAN (Figure 3) were mostly similar to the trends in their concentrations i.e. decreasing amounts of C and N with increasing grazing pressure. The exception was SAN, which increased with increasing grazing pressure in the 0–10 cm layer. The overall trend for SOC, STN, and SAN was a decline at each successive depth increment from 0 to 50 cm depth, except SAN, which was lower at 0–10 cm than 10–20 cm for the UG, LG, and MG. SOC differed significantly for the overall 0–50 cm profile decreasing from UG through LG and MG to HG (Figure 3A). The UG plots had significantly higher STN levels (1.69 kg m^−2^), HG plots were generally the lowest (1.22 kg m^−2^) and LG and MG were intermediate with no significant difference (Figure 3B). HG plots generally had the lowest SAN (644 kg hm^−2^) while the other grazing intensities did not differ for the overall 0–50 cm profile (Figure 3C). The C:N ratio across the entire soil profile was significantly greater for the LG pasture (9.74) compared to UG (7.71), MG (8.22) and HG pasture (7.93) (Figure 3D).

## Discussion

### Vegetation characteristics

Our results indicate that vegetation height, canopy cover, and plant species abundance decreased significantly with increased grazing intensities. This change in plant species abundance in our study is consistent with a study conducted in Inner Mongolia [33], which showed reduced species richness of the *Aneurolepidium chinense* and *Stipa grandis* steppes with an increase in grazing intensity.

In our study, aboveground biomass decreased significantly with increased grazing intensity, which is not consistent with research from alpine meadow in the eastern Tibetan Plateau. Gao et al. [34] examined the seasonal dynamics of biomass and plant nitrogen content under light, moderate, and heavy grazing intensities and found that the highest live aboveground biomass occurred at sites with moderate grazing pressure. Possible explanations for heavy grazing reducing aboveground biomass include the decrease in abundance of the initially dominant and palatable graminoid species (e.g. *Leymus chinensis* and *Stipa grandis*) with increasing grazing intensity and the subsequent domination of unpalatable forbs and legumes became dominant. Likewise, the reduced canopy cover caused by selective grazing decreasing the area and resources available for plant production probably contributes to this change[3].

Our results showed that change of belowground biomass was different from that of aboveground biomass. These changes were not consistent with the findings of Su et al. [35], who observed that steppe vegetation grows better, produces more root exudates and develops a stronger root system in un-grazed areas compared to grazed areas in the semi-arid region of northern China. Other studies also showed variable findings for belowground biomass affected by grazing, such as an increase [34], decrease [36], or no effect[37]. In our grazing region, these results indicate that proper grazing intensity would stimulate vegetation growth in *Leymus chinensis* steppe, and overgrazing would decrease the steppe productivity.

### Soil moisture content

Grazing has significant impacts on soil water through its influence on infiltration via treading and altering evapo-transpiration through defoliation[38] [39], i.e. soil water content declines under poor infiltration and/or high evapo-transpiration [38]. Our study showed soil water content decreased with increasing grazing intensity, consistent with previous research conducted to quantify the water fluxes as affected by grazing intensity in Inner Mongolia Grassland of northern China [40]. This is likely associated with a reduction in vegetation height, canopy cover, and biomass as grazing intensity increased. The poor ground cover directly contributed to the increase of soil evapo-transpiration loss. Larger vegetation and litter biomass remaining in the low grazing intensity, compared to heavier grazing, would increase the holding capacity of surface soil water [38].

### Soil bulk density

Increased bulk densities and lower soil moisture content, as a result of increased animal trampling, have been observed for different grazing animals in different grassland ecosystems [41]. Our results were consistent with previous research conducted on the Loess Plateau that showed grazing significantly increased soil bulk density and decreased soil moisture content[41]. He et al. [18] also found that bulk density of temperate grasslands in northern China increased linearly with increasing stocking rates in both the 0–10 cm and 10–30 cm soil layers.

### SOC, STN, and SAN

Grazing exclusion can enhance soil C and N storage in temperate grasslands in northern China[18]. Reduction in soil C and N storage under long-term heavy grazing was reported previously from research carried out in this region [9] [18] [20]. Our results were consistent with previous studies. The response of SOC, STN, and SAN storage to different grazing intensities in our study may be due, in part, to the effects of grazing on belowground biomass, litter and standing dead components of the aboveground biomass. Belowground biomass has been proven to be positively correlated with the size of the soil organic matter pool[42]. Repeated and frequent grazing resulted in decreased root elongation and biomass, and hence lower C inputs into the soil from the roots[43]. Livestock grazing altered plant composition, canopy cover, and community biomass. This may account for changes in the storage of SOC, STN, and SAN in the soil under different grazing intensities.

Possible explanations for overgrazing reducing SOC storage include vegetation production reduction, vegetation destruction, and change of environmental factors[1]. Grazing might also be expected to influence the organic C content of the soil by reducing vegetation growth, the amount of litter and exposing the soil surface to erosion, which leads to direct soil nutrient losses [39]. Disruption of soil aggregate structure and surface soil crust due to trampling increases the decomposition of soil organic matter and renders the soil susceptible to water and wind erosion[44]. In temperate grasslands, considerable loss of SOC and soil N is caused by wind and water erosion, particularly in areas with sandy soil and high wind speed [45]. Soils under HG in this region, with lower canopy cover and lower vegetation height, are vulnerable to wind erosion[21].

In the current study, a similar concentration of available N was found among UG, LG and MG plots, maybe due to organic matter and nutrient transfer via animal excreta[46], although there were significant differences in aboveground and belowground biomass among these plots. But defoliation in HG significantly decreased both STN and SAN by decreasing root and total production, resulting in reduced soil C and N [47]. The changes in plant community composition due to selective foraging can also indirectly influence soil N and available N by affecting plant litter decomposition rates and soil microbial activities [48].

The higher C:N ratio in LG suggests either a slower rate of decomposition or a greater portion of recent plant material in the residue[1]. The higher N content in the UG suggests that grazing reduced soil N [1]. A lower C:N ratio in UG perhaps means that higher soil moisture is stimulating litter decomposition, thereby releasing more N. Lower C:N ratios in MG and HG are perhaps because grazers return large amounts of N to the soil through urine and feces, increasing levels of available soil N [49]. However, grazing can also reduce N turnover and availability as grazers feed selectively on plants with high N content and thus increase the dominance of plant species with low N content[50].

## Conclusions and Implications

Our study revealed that vegetation characteristics and soil C and N storage of semiarid grassland in the agro-pastoral zone were sensitive to grazing practice. With increasing grazing intensity, the vegetation height, canopy cover, plant species abundance and aboveground biomass decreased significantly. In addition, ten years of grazing not only changed the distributions of C and N through the soil profile (0–50 cm), but also the total storages of C and N in the soil profile. As a result, SOC and STN in the 0–50 cm were highest under UG (13.3 kg C m^−2^ and 1.69 kg N m^−2^) and lowest under HG (9.8 kg C m^−2^ and 1.22 kg N m^−2^). SAN was significantly lower under HG (644 kg N hm^−2^) than under other treatments (725–731 kg N hm^−2^) in the 0–50 cm.

Our results indicated that the pasture management of “take half-leave half” has potential benefits for primary production and livestock grazing, which would achieve a balance between protection of species diversity, livestock production and soil C and N management in this region. However, the “Reduce Livestock Return the Grasslands” program with grazing exclusion supported by the Ministry of Agriculture of the People's Republic of China in 2002 was perhaps the effective choice for restoring degraded grasslands in the agro-pastoral zone. Therefore, flexible rangeland management should be adopted that suits local circumstances by balancing the demand for grassland utilization and conservation in this region. These results would be useful to the decision making on rangeland management in terms of maximizing C and N sequestration while maintaining adequate productivity for servicing the regional socio-economic development of the agro-pastoral zone in northern China.
